# Utilization Trends of Novel Hormonal Agents in Metastatic Castration-Resistant Prostate Cancer in Quebec

**DOI:** 10.3390/curroncol29110680

**Published:** 2022-11-12

**Authors:** Jason Hu, Armen G. Aprikian, Ramy R. Saleh, Alice Dragomir

**Affiliations:** 1 Division of Urology, Department of Surgery, McGill University, Montreal, QC H3A 0G4, Canada; 2 Department of Oncology, McGill University, Montreal, QC H3A 0G4, Canada; 3 Division of Urology, McGill University Health Centre, Montreal, QC H4A 3J1, Canada; 4 Division of Medical Oncology, McGill University Health Centre, Montreal, QC H4A 3J1, Canada

**Keywords:** prostate cancer, abiraterone, enzalutamide, drug utilization

## Abstract

Background: The introduction of novel hormonal agents (NHAs) such as abiraterone acetate (ABI) and enzalutamide (ENZ) for metastatic castration-resistant prostate cancer (mCRPC) was an important milestone given their survival benefits, tolerability, and ease of administration relative to taxane chemotherapies. This descriptive study sought to describe the utilization trends of ABI and ENZ in patients with mCRPC in the early years after their approval in the province of Quebec in Canada. Methods: A retrospective population-based cohort was extracted from Quebec public healthcare administrative databases. The cohort included first-time users of NHAs (ABI or ENZ) from 2011 to 2016. The primary analyses aimed to describe the overall temporal trends (2011–2016) of NHA initiators by chemotherapy status (chemotherapy-naïve versus post-chemotherapy), and prescribing specialty (medical oncology versus urology versus others). Results: The cohort comprised 2183 patients, with 1562 (72%) in the chemotherapy-naïve group and 621 (28%) in the post-chemotherapy group. While the majority of patients were post-chemotherapy NHA initiators in 2012, this proportion decreased over time and accounted for only 13% of NHA initiators by the end of 2016. Medical oncologists were the most frequent prescribers of NHAs (upwards of 60%) throughout 2012 but fell to 45% by the end of 2016. Conversely, the proportion of prescriptions by urologists increased from 22% in 2012 to 42% in 2016. Conclusion: Over time, there was an increasing proportion of (1) patients who initiated NHAs without prior chemotherapy treatment, (2) NHA prescribing by urologists, and (3) ENZ users. Taken together, this implies that the introduction of NHAs has altered the management of mCRPC and urologists quickly adopted NHAs into their practice.

## 1. Introduction

Prostate cancer (PCa) is the most common noncutaneous malignancy in Canadian men [1]. Most patients are diagnosed at the localized stage and the survival rate is high [2]. However, certain patients will progress to metastatic castration-resistant prostate cancer (mCRPC) which is defined as progression despite castrate levels of testosterone [3,4]. Until 2010, docetaxel chemotherapy was the only treatment with survival benefit in this disease setting. Although it remains an ultimately incurable disease, the treatment landscape for mCRPC has seen significant advances in the past decade [5]. Progress was made with the introduction of the second-generation taxane chemotherapy and cabazitaxel, and further innovation was represented with immunotherapeutic and radiopharmaceutical agents in the form of sipuleucel-T and radium-223, respectively. However, the most important additions have been the novel hormonal agents (NHAs) which include abiraterone acetate (ABI) and enzalutamide (ENZ); their adoption was rapid and in greater magnitude than the other novel mCRPC treatments [6]. This could be attributed to several reasons: their approval of both in pre- and post-docetaxel settings in mCRPC, their ease of delivery (oral administration), and their tolerability compared to taxanes [5].

In their respective pivotal clinical trials leading to their regulatory approval, ABI and ENZ demonstrated similar survival benefits relative to their placebo control arms [7,8,9,10]. In the COU-AA-301 trial which randomized post-docetaxel mCRPC patients to either ABI or placebo-prednisone, overall survival was longer in the ABI group (ABI: 14.8 months vs. placebo: 10.9 months; hazard ratio: 0.65, *p* < 0.001) [7]. In the AFFIRM trial which randomized post-chemotherapy mCRPC patients to either ENZ or placebo, ENZ also improved overall survival (ENZ: 18.4 months vs. placebo: 13.6 months; hazard ratio: 0.63, *p* < 0.001) [8]. In the COU-AA-302 trial, chemotherapy-naïve mCRPC patients were randomized to either ABI or placebo-prednisone and ABI outperformed placebo again in terms of overall survival (ABI: median not reached vs. placebo: 27.2 months; hazard ratio: 0.75, *p* = 0.01) [9]. Similarly, the PREVAIL randomized trial enrolled chemotherapy-naïve mCRPC patients and found that ENZ improved overall survival compared to placebo (ENZ: 32.4 months vs. placebo: 30.2 months, hazard ratio: 0.71, *p* < 0.001) [10]. To date, no head-to-head randomized trial of ABI and ENZ sufficiently powered for detecting differences in overall survival in mCRPC have been conducted.

While the two have an overall favorable toxicity profile, the adverse effects associated with each are different owing to their specific mechanism of action. Abiraterone acetate is an androgen-biosynthesis inhibitor and should be co-administered with prednisone, while ENZ is a second-generation competitive inhibitor of the androgen receptor [11,12]. Specifically, ABI is a selective irreversible inhibitor of CYP17, an enzyme necessary for the production of extragonadal and testicular androgens [11]. The required co-administration of prednisone with ABI is to counteract the rise in adrenocorticotropic hormone caused by the decrease in cortisol production resulting from blocking CYP17. On the other hand, ENZ competitively binds to the ligand-binding domain and inhibits the following aspects of the androgen receptor: nuclear translocation of the androgen receptor, recruitment of cofactors, and binding to DNA [12]. Adverse effects associated with ABI include liver function abnormalities, fluid retention, and cardiac events [13], whereas ENZ is associated with more central-nervous-system impairments and hot flashes [14]. Increased incidence of hypertension and fatigue are reported with both of these agents [5].

In the province of Quebec in Canada, ABI was approved for public reimbursement in 2012 for patients with mCRPC previously treated with docetaxel chemotherapy. However, patients ineligible for chemotherapy could still have access to the drug on an exceptional medical basis. In 2014, ABI was also approved for patients with mCRPC without prior exposure to docetaxel chemotherapy. For ENZ, its initial approval in the post-docetaxel setting came in 2014. As with ABI in 2012, patients ineligible for chemotherapy could also have access on an exceptional medical basis. The subsequent approval of ENZ in mCRPC patients without prior chemotherapy occurred in 2016.

There are currently limited data related to the adoption of these NHAs in clinical practice outside of the United States, and none in Canada. While urologists are heavily involved in the management of localized disease and also frequently prescribe androgen-deprivation therapy (ADT) for metastatic disease, the management of mCRPC has generally been reserved for medical oncologists due to the sole available treatment with survival benefit being docetaxel chemotherapy. The arrival of NHAs has potential implications in altering this dynamic. With expanding therapeutic options and new treatment paradigms in advanced PCa, it is important to examine the prescribing patterns of these NHAs among patients with mCRPC over this initial period of approval to assess how they are being used in routine clinical practice. In this study, we sought to describe the utilization trends of the NHAs in the province of Quebec in Canada.

## 2. Materials and Methods

### 2.1. Data Sources

As with other Canadian provinces, in Quebec, provincial public healthcare insurance coverage is provided to all its residents for physician visits and medical procedures. This study draws data from public healthcare administrative databases from the province of Quebec, which are administered by the Régie de l’assurance maladie du Québec (RAMQ). The RAMQ provides universal healthcare coverage to residents of the province of Quebec in Canada through the Quebec Health Insurance Plan. This plan covers all physician visits and procedures, and outpatient and inpatient care for all Quebec residents. The prescription drug insurance plan of the RAMQ (Public Prescription Drug Insurance Plan) provides coverage for individuals aged 65 years and older, welfare recipients, and other residents who do not have access to a private drug insurance plan. The RAMQ databases contain data pertaining to basic patient demographic information, medical services derived from physician billing claims, and prescription drugs dispensed at community pharmacies. Data on hospital admissions were extracted from a complementary source, the Maintenance et exploitation des données pour l’étude de la clientèle hospitalière (MED ECHO) databases.

### 2.2. Study Cohort

This was designed as a retrospective observational study with a cohort composed of men who initiated an NHA (ABI or ENZ) in the period from January 2011 to December 2016. Patients with no prior history of ADT (luteinizing hormone-releasing hormone agonist or antagonist drugs or orchiectomy) and who were registered to the public drug insurance plan for less than a year prior to NHA initiation were excluded. The index date corresponded to the date of the first prescription of the NHA. It should be noted that although, nowadays, both drugs have gained expanded approval to earlier disease states, at the time, both ABI and ENZ were considered “exception drugs” by the RAMQ and were only approved for mCRPC during the study period in the province of Quebec. This ensured that NHAs received by patients in the study were for the treatment of mCRPC.

### 2.3. Primary Analyses

The primary analyses aimed to describe the overall temporal trends (2011–2016) of NHA initiators by chemotherapy status (chemotherapy-naïve versus post-chemotherapy), and prescribing specialty (medical oncology versus urology versus others). Patients were considered as post-chemotherapy if physician claims of intravenous chemotherapy administration were identified in the period between ADT initiation and NHA initiation. The prescribing specialty was based on the physician specialty identified in the initial NHA prescription and was obtained from the prescription-drug database. The medical oncology grouping included both medical oncologists and hematologists. Physician specialties other than urology and medical oncology (medical oncologists and hematologists) were grouped as others.

### 2.4. Secondary Analyses

As part of secondary analyses, we sought to describe the trends for each NHA separately in the years when both ABI and ENZ were available (2014 to 2016, referred to hereafter as the ENZ-era). Specifically, we examined the evolution of patterns by chemotherapy status and prescribing specialty within each NHA type.

### 2.5. Patient Characteristics

Patient characteristics such as age and region of residence (urban vs. rural) were captured at the index date. Characteristics relating to PCa included: prior local PCa treatment (receipt of radical prostatectomy, external beam-radiotherapy, or brachytherapy at any time prior to the index date), time from PCa diagnosis to index date, use of bone-targeted therapy (zoledronic acid or denosumab in the year prior to the index date), chemotherapy status (chemotherapy-naïve vs. post-chemotherapy), and symptomatic indicator (yes vs. no). The symptomatic indicator variable was meant to be a proxy of patient symptomatic status and was a composite variable of the any of the following conditions identified in the 3 months prior to the index date: receipt of a urological procedure relating to loco-regional complications of PCa (e.g., nephrostomy or urethral stenting), receipt of palliative radiotherapy, or use of opiates. All other comorbidities, the Charlson comorbidity index, and healthcare utilization metrics (hospitalization and visits to specialist physicians) were measured during the year prior to the index date through diagnosis codes and treatments contained in physician claims, inpatient discharge abstracts, and prescription-drug databases.

### 2.6. Statistical Analysis

Descriptive statistics were presented as counts and percentages for categorical variables, and as means with standard deviation for continuous variables. Temporal trends of NHA initiation were observed in tri-monthly intervals (quarters: Q1, Q2, Q3, and Q4) over the study years. Temporal trends were evaluated with either the Cochran–Armitage test or the Cochran–Mantel–Haenszel test, whichever was appropriate in a given analysis.

Multivariable logistic regression analyses were performed to identify factors associated with the initiation of ENZ over ABI in the ENZ-era with the main goal of assessing the effect of the prescribing-specialty variable when adjusted for other baseline patient characteristics. Two models were fitted, with one model for chemotherapy-naïve patients and the other for post-chemotherapy patients. Results from the multivariable models are presented as odds ratios (OR) with 95% confidence intervals (95% CI). All analyses were two-sided with the statistical significance level set at *p* < 0.05, and were conducted with SAS software version 9.4 (SAS Institute, Cary, NC, USA).

### 2.7. Sensitivity Analyses

To account for potential discrepancies in the initial prescribing physician and subsequent specialties prescribing the NHA for a given patient, we also repeated analyses with an alternative definition for the prescribing specialty. In this alternative definition, the prescribing specialty was defined as the specialty accounting for the majority of the NHA prescriptions for a given patient. In another sensitivity analysis, we classified radiation oncologists along with the urologists and repeated the analyses involving prescribing specialties (medical oncologists vs. urologists/radiation oncologists vs. others).

## 3. Results

The study cohort comprised 2183 patients who initiated an NHA during the study period. These patients filled a total of 29,347 NHA prescription claims and the absolute number of claims increased yearly (2011: 133; 2012: 1958; 2013: 4172; 2014: 6767; 2015: 7796; and 2016: 8521). Additional descriptive claims-level details can be found in the Supplementary Table S1.

### 3.1. Baseline Characteristics

Table 1 displays the baseline characteristics of NHA users stratified by chemotherapy status. Chemotherapy-naïve patients tended to be older and have a greater number of comorbidities relative to post-chemotherapy patients. On the other hand, post-chemotherapy patients had higher proportions for the variables relating to PCa severity (symptomatic indicator, use of bone-targeted therapy), a shorter time from PCa diagnosis to the index date, and a higher number of visits to specialist physicians in the year before starting the NHA.

### 3.2. Primary Analysis: Overall Trends of Chemotherapy Status and Prescribing Specialties (2011–2016)

**Figure 1:**
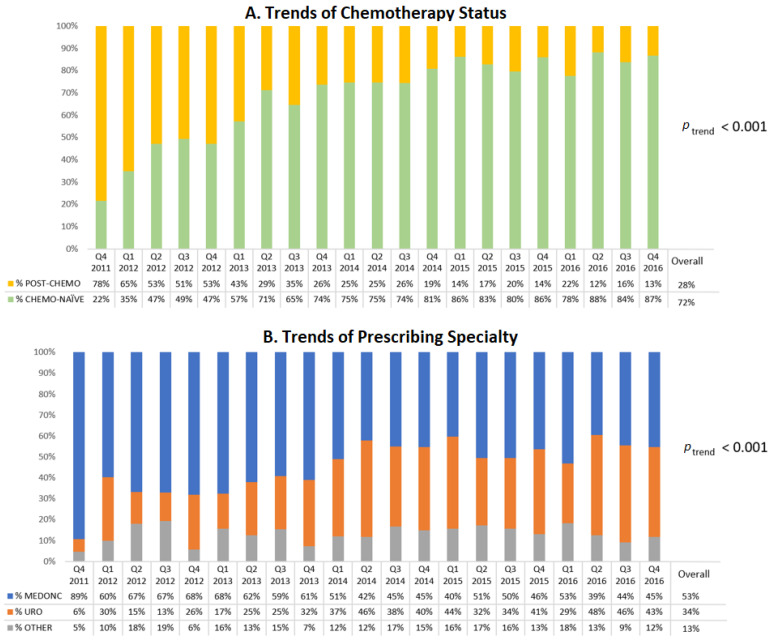
Overall temporal trends in initiation of novel hormonal agents from 2011 to 2016. (**A**) Stratified by chemotherapy status. (**B**) Stratified by prescribing specialty.

Figure 1A displays the trend of NHA initiators by chemotherapy status. During the first year of the study, the majority of NHA users were previously treated with chemotherapy but this proportion decreased over time and accounted for only 13% of NHA users in the last quarter of 2016 (*p* < 0.001). This decreasing trend of post-chemotherapy NHA initiators (and conversely the increasing trend of chemotherapy-naïve patients) was observed in both the ABI users (*p* < 0.001, Supplementary Figure S1) and ENZ users (*p* < 0.001, Supplementary Figure S2).

Medical oncologists represented the most frequent prescribers of NHAs (upwards of 60%) throughout 2012 but fell to 45% by the end of 2016 (Figure 1B). Conversely, the proportion of prescriptions by urologists increased from 22% in 2012 to 42% in 2016 (Figure 1B).

### 3.3. Secondary Analyses: Trends in the ENZ-Era (2014–2016)

**Figure 2:**
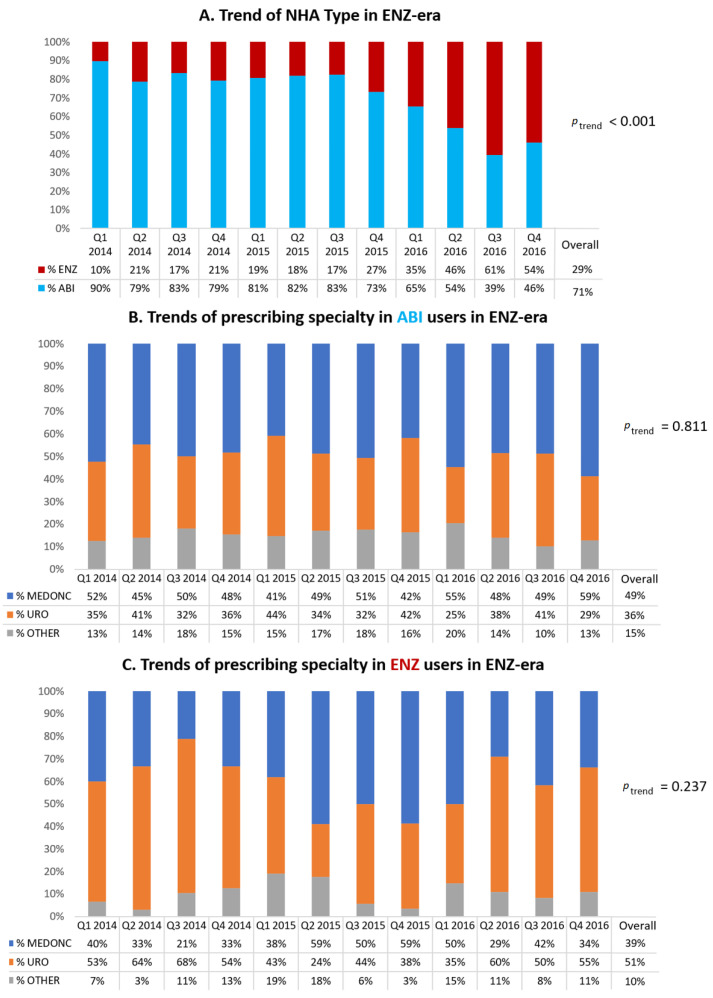
Temporal trends of novel hormonal agent (NHA) initiators in ENZ-era (2014–2016). (**A**) Trends of NHA type. (**B**) Trends of prescribing specialty in ABI users. (**C**) Trends of prescribing specialty in ENZ users.

Figure 2A displays the trends of NHA type in the ENZ-era. From its first quarter of approval (Q1 of 2014), the proportion of ENZ initiators increased from 10% to 54% at the end of the study period (*p* < 0.001).

Regarding ABI initiators in the ENZ-era (Figure 2B), there was a slight majority of medical oncologists as prescribers (accounting for 49% overall during those years) with urologists ranking second at 36%. For ENZ initiators in the ENZ-era (Figure 2C), it was the opposite, with a slight majority of urologists as prescribers (accounting for 51% overall during those years) and medical oncologists coming in at second at 39%.

**Figure 3:**
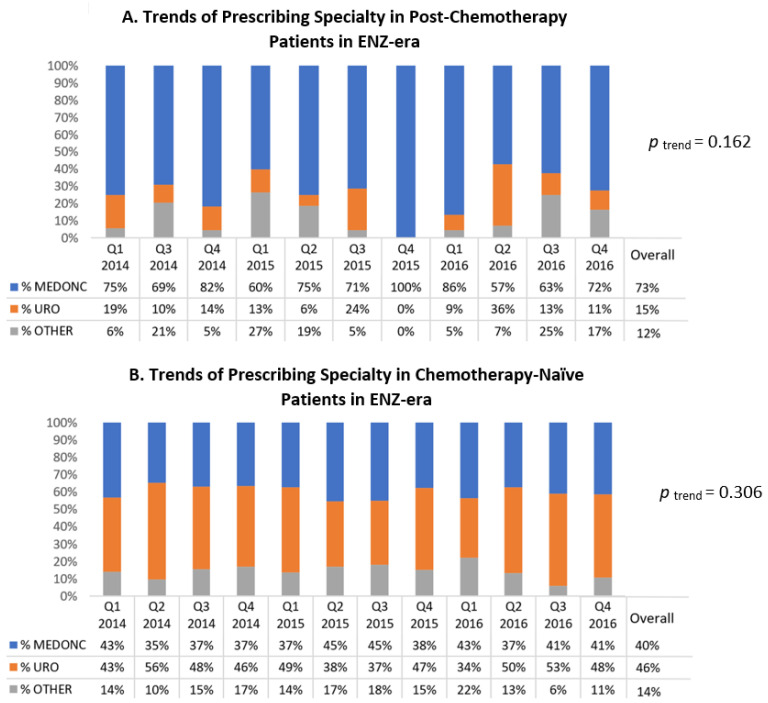
Trends of prescribing specialty of novel hormonal agent (NHA) initiators stratified by chemotherapy status in ENZ-era. (**A**) Trends of prescribing specialty among post-chemotherapy patients. (**B**) Trends of prescribing specialty among chemotherapy-naïve patients.

Among post-chemotherapy patients in the ENZ-era (Figure 3A), medical oncologists were the most frequent prescribing specialty throughout those years (accounting for 73% overall during the ENZ-era). Additionally, ABI was the NHA used by the majority of these patients up until the second quarter of 2016 (Supplementary Figure S3).

Among chemotherapy-naïve patients in the ENZ-era (Figure 3B), urologists were the top prescribing specialty accounting for 46% of NHA prescribers in the period and medical oncologists ranked second at 40%. These percentages remained relatively stable throughout (temporal trend *p* = 0.306). Regarding the type of NHA used in these patients, the majority of patients were treated by ABI up until the third quarter of 2016 (Supplementary Figure S4).

In Table 2, multivariable regression analyses examining the factors associated with initiating ENZ (over ABI) during the ENZ-era confirmed that urologists were likely to prescribe ENZ compared to medical oncologists in both the chemotherapy-naïve setting (Model 1, OR 1.89, 95% CI 1.38–2.58) and the post-chemotherapy setting (Model 2, OR 3.83, 95% CI 1.76–8.36). Other statistically significant variables found in the chemotherapy-naïve setting included later years of initiation (OR_2015_ 1.57, 95% CI 1.06–2.33; OR_2016_ 6.89, 95% CI 4.81–9.87), age ≥75 (OR 1.38, 95% CI 1.01–1.88), rural residence (OR 1.54; 95% CI 1.13–2.07), prior use of bone-targeted therapy (OR 1.49; 95% CI 1.08–2.03), and ≥1 pre-existing one cardiovascular condition (OR 1.54; 95% CI 1.06, 2.25). In the post-chemotherapy model, later year of initiation was a statistically significant variable (OR_2016_ 2.25, 95% CI 1.13–4.47).

### 3.4. Sensitivity Analyses

Given that the initial prescribing specialty concorded with the alternative definition of prescribing specialty (specialty prescribing the majority of the NHA for a given patient) in 94% of patients, analyses repeated with that definition gave quasi-identical results (results not shown). Similarly, the proportion of patients who had their NHA prescribed by radiation oncologists was minimal (2.8%). The repeated analyses with the combined urology/radiation oncology grouping were essentially the same as the original grouping (results not shown).

## 4. Discussion

This descriptive study examined the prescribing trends of NHAs in the early years of approval in the province of Quebec in Canada. Over time, there was an increasing proportion of patients who initiated NHAs without prior chemotherapy treatment, of NHA prescribing by urologists, and of ENZ users.

To the best of our knowledge, this is the first report on the utilization patterns of NHAs in mCRPC in Canada. A previous study examining the adoption of ABI and ENZ in the United States corroborates some of our findings [15]. They noted that ENZ had become the most prescribed NHA by 2016, which is similar to our results of ENZ accounting for slightly over 50% of NHA initiators in the latter half of 2016. A Swedish study also noted that more patients were prescribed with ENZ over ABI in 2015–2016 [16].

From one perspective, these results may seem counterintuitive if one expects that ABI should have maintained some dominance as a preferred choice over ENZ given it was approved first. In that line of thought, the two-year gap between the introduction of these two agents in the province should have led to some familiarity and physician preference for ABI. Our results suggest this was not the case. To our knowledge, clinical guidelines do not favor either ABI or ENZ in mCRPC [17,18,19]. Most guidelines recommend that the specific choice of NHA should come down to comorbidities and patient and physician preference. These two drugs, although relatively tolerable, possess different adverse-event profiles. The overall rapid adoption of ENZ may also be related to some constraints with ABI: the necessary concomitant use of prednisone and the need for monthly monitoring of blood pressure, potassium, and liver function [13]. These constraints could make ENZ the preferable choice for physicians and patients alike. On a related note, in regression modeling, we identified rural region of residence as a predictor for ENZ over ABI prescription (albeit only for chemotherapy-naïve patients). One possible explanation could be that patients living further away from cancer-treatment centers or clinics may prefer the treatment that does not require as much stringent monitoring.

Our findings support the notion that urologists are increasingly prescribing NHAs. This was also reflected in previous research, where it was specifically noted that the number of moderate-to-high-volume ABI-prescribing urologists tripled from 2013 to 2016 while the corresponding trend in moderate-to-high-volume ABI-prescribing non-urologists was only a 30% relative increase [15]. An even more dramatic 3000% growth in moderate-to-high-volume ENZ-prescribing urologists was observed during that period. In another study using American data, it was found that while the majority of NHA prescriptions originate from medical oncologists, the proportion of prescriptions by urologists has doubled for ABI and tripled for ENZ [20].

Beyond the increase in the number of urologist prescribing, the aforementioned figures from these two previous studies also suggest a preference for ENZ among urologists [15,20]. This is further confirmed by another study demonstrating that ENZ is more likely to be prescribed by urologists than by medical oncologists, which is also consistent with our results [21]. As mentioned earlier, there are several constraints that come with ABI prescribing, and they may present a stronger disincentive to urologists relative to medical oncologists. Compared to a typical medical oncology practice, a typical urology practice may not be as suited to manage frequent monitoring of blood pressure and potassium and liver function as well as potential issues with prednisone treatment.

We found that the proportion of NHA initiators who were not previously treated with chemotherapy increased rapidly. In ABI users, this change happened even before its approval for use in chemotherapy-naïve patients occurred in 2014; in fact, the majority of ABI users were chemotherapy-naïve patients by 2013. For ENZ, the majority of users were chemotherapy-naïve patients from the onset of approval. These findings suggest that overall, more mCRPC patients are receiving life-prolonging treatment. Prior to the advent of NHAs, docetaxel chemotherapy was the only recourse but its uptake was always limited due to the high proportion of mCRPC patients being either too frail for treatment or due to patient preference [22,23]. As the baseline characteristics of our chemotherapy-naïve group show, they are on average nearly 5 years older than the post-chemotherapy patients when initiating an NHA.

Taken altogether, our findings advance the notion that care pathways for mCRPC may have changed with the introduction of NHAs as urologists are increasingly prescribing these life-prolonging treatments. Traditionally, urologists would refer patients with mCRPC to medical oncologists for chemotherapy administration. With the NHAs, urologists have the possibility to be more involved in the management of advanced PCa patients, and for longer. This effect may become even sharper with the expanding disease settings where NHAs have shown benefits (metastatic castration-sensitive PCa (mCSPC) and non-metastatic CRPC (nmCRPC)) and the approval of more NHAs (apalutamide and darolutamide). This has the potential beneficial implication of better continuity of care for patients as urologists typically have already followed a PCa patient for many years until the point of mCRPC.

## 5. Limitations

As these findings only represent the early approval period of NHAs, further follow-up is required to confirm if these patterns persist over time. This limitation is particularly notable for ENZ, as we only captured its initial two years of approval in the provincial drug plan. Furthermore, given the time frame of this dataset, we could not study the utilization of these NHAs in earlier disease settings (mCSPC or nmCRPC) or the additional impact of newer NHAs.

While the use of administrative healthcare data allows for a representative portrait of the use of these treatments in clinical practice, several limitations exist with these data. Several clinical- and disease-related variables of interest are not captured in these databases, such as cancer staging, Gleason grading, prostate-specific antigen serum levels, metastatic burden, functional status, and presence of symptoms. However, we did use proxy variables (symptomatic indicator, use of bone-targeted therapy, time from PCa diagnosis to index date, etc.), to partially remediate this issue in an attempt have some reflection of cancer severity and symptomatic status.

A further limitation concerns the identification of chemotherapy regimens. Through physician claims data, we can identify the procedure act of intravenous chemotherapy administration, however the identification of the actual chemotherapy regimen used is not available in our datasets. Consequently, we could not identify, with certainty, if the chemotherapy regimen was docetaxel. However, given the study period, docetaxel was the only chemotherapy with survival benefits in PCa, as cabazitaxel was not yet approved in the provincial drug insurance plan. Furthermore, we only considered chemotherapy cycles that were started during the period from ADT initiation to NHA initiation.

## 6. Conclusions

The introduction of NHAs in mCRPC represented a critical landmark for patients as they were the first oral drugs offering survival benefit in this disease setting. Although ABI was introduced earlier than ENZ, the uptake of ENZ was relatively rapid and by the end of the study period, both NHAs were equally used. Along with this rapid adoption of ENZ, the proportion of NHAs prescribed by urologists increased over the years. Over time, the majority of patients who initiated NHAs were chemotherapy-naïve. Finally, our findings also suggest that disease management for advanced PCa may have changed as urologists seem to maintain a more prominent role even in mCRPC. Further research examining how exactly the introduction of NHAs has impacted disease management and referral patterns in advanced PCa may be of interest to clinicians and policy-makers.

## Figures and Tables

**Table 1:** Baseline characteristics.

	Chemotherapy-Naïve (*n* = 1562)	Post-Chemotherapy (*n* = 621)
**Characteristic**		
Age, years; mean (SDev)	78 (8.1)	73 (8.0)
Rural	32.2	33.8
Time from PCa dx to index, years; mean (SDev)	8.1 (5.6)	7.2 (5.1)
Prior local PCa treatment	39.1	43.6
Symptomatic indicator	41.1	52.0
Bone-targeted therapy	34.2	71.0
Charlson index ≥ 4	6.7	5.6
Hypertension	70.5	67.2
Dyslipidemia	57.2	53.1
Diabetes	22.8	20.6
Coronary artery disease	14.5	14.2
Myocardial infarction	3.1	2.6
Cerebrovascular disease	2.8	1.3
Heart failure	4.9	2.9
Arrhythmia	12.5	9.0
Peripheral artery disease	5.1	4.8
Venous thromboembolism	2.9	4.4
≥1 cardiovascular condition	28.1	25.8
Renal disease	9.9	7.7
Liver disease	1.7	0.7
Nbr SPC visits; mean (SDev)	13.8 (11.6)	22.0 (11.0)
Hospital admission (≥1)	41.8	45.7
Nbr drug classes; mean (SDev)	12.8 (5.2)	14.5 (5.2)
Novel hormonal agent		
ABI	78.8	87.3
ENZ	22.2	12.7
Prescribing specialty		
Medical oncologist	44.5	74.4
Urologist	41.6	14.7
Other	14.0	11.0

All numbers represent percentages unless otherwise noted. Abbreviations: Nbr = number; PCa = prostate cancer; SDev = standard deviation; SPC = specialist physician.

**Table 2:** Multivariable analysis of variables associated with receiving ENZ (over ABI) in ENZ-era.

	Model 1: CHEMOTHERAPY-NAÏVE (*n* = 1128)				Model 2: POST-CHEMOTHERAPY (*n* = 263)			
FACTORS	OR	95% CI		*p*-Value	OR	95% CI		*p*-Value
Prescribing specialty (Ref: Medical oncology)		-				-		
Urology	1.89	1.38	2.58	<0.001	3.83	1.76	8.36	<0.001
Other	0.80	0.49	1.28	0.350	1.13	0.45	2.82	0.791
Year of initiation (Ref: 2014)		-				-		
2015	1.57	1.06	2.33	0.023	0.93	0.45	1.94	0.859
2016	6.89	4.81	9.87	<0.001	2.25	1.13	4.47	0.022
Age (Ref: <75)		-				-		
≥75	1.38	1.01	1.88	0.041	1.54	0.81	2.90	0.180
Residence (Ref: Urban)		-				-		
Rural	1.54	1.13	2.07	0.005	0.67	0.35	1.24	0.205
Symptomatic indicator (Ref: No)		-				-		
Yes	0.94	0.70	1.26	0.688	0.93	0.52	1.69	0.834
Prior Bone-targeted therapy (Ref: No)		-				-		
Yes	1.49	1.08	2.03	0.013	1.00	0.53	1.87	0.992
Prior local treatment (Ref: No)		-				-		
Yes	0.86	0.64	1.17	0.341	0.66	0.35	1.24	0.201
Charlson comorbidity index (Ref: <4)		-						
≥4	1.09	0.61	1.97	0.752	0.68	0.16	2.89	0.612
Hypertension (Ref: No)		-				-		
Yes	0.78	0.55	1.11	0.169	1.47	0.73	2.96	0.269
Hyperlipidemia (Ref: No)		-						
Yes	1.18	0.86	1.63	0.286	1.18	0.61	2.29	0.610
Diabetes (Ref: No)		-						
Yes	1.30	0.91	1.84	0.149	1.13	0.55	2.31	0.746
Cardiovascular condition (Ref: 0)		-						
≥1	1.54	1.06	2.25	0.022	0.65	0.32	1.35	0.257

Abbreviations: ABI = abiraterone acetate; CI = confidence interval; ENZ = enzalutamide; OR = odds ratio; Ref = reference.

## Data Availability

The authors do not have permission to share the data extracted from the Quebec Health Insurance Board (RAMQ) database for this study. Data requests must be made directly to the RAMQ.

## Supplementary Materials

The following are available online at https://www.mdpi.com/article/10.3390/curroncol29110680/s1, Figure S1: Trend of chemotherapy status in ABI users, Figure S2: Trend of chemotherapy status in ENZ users, Figure S3: Trend of NHA type in post-chemotherapy patients in ENZ-era, Figure S4: Trend of NHA type in chemotherapy-naïve patients in ENZ-era, Table S1: Description of novel hormonal agent prescription claims.
