# Supplementary material for: Utilization Trends of Novel Hormonal Agents in Metastatic Castration-Resistant Prostate Cancer in Quebec
Source: Curr Oncol. 2022 Nov 12;29(11):8626–37. doi: 10.3390/curroncol29110680 (PMC9688923; doi:10.3390/curroncol29110680)
Supplement: Supplementary file 1 [file curroncol-29-00680-s001.zip › curroncol-1928558-supplementary.pdf]

Supplementary Materials

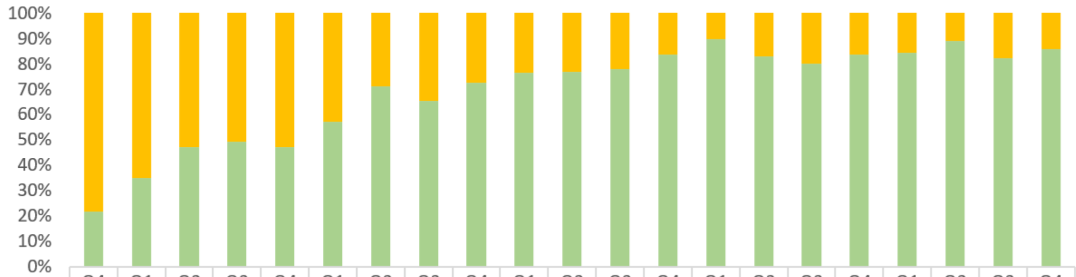

	Q4 2011	Q1 2012	Q2 2012	Q3 2012	Q4 2012	Q1 2013	Q2 2013	Q3 2013	Q4 2013	Q1 2014	Q2 2014	Q3 2014	Q4 2014	Q1 2015	Q2 2015	Q3 2015	Q4 2015	Q1 2016	Q2 2016	Q3 2016	Q4 2016
% POST-CHEMO	78%	65%	53%	51%	53%	43%	29%	35%	28%	23%	23%	22%	16%	10%	17%	20%	16%	16%	11%	18%	14%
% NO-CHEMO	22%	35%	47%	49%	47%	57%	71%	65%	72%	77%	77%	78%	84%	90%	83%	80%	84%	84%	89%	82%	86%

Figure S1. Trend of chemotherapy status in ABI users,.

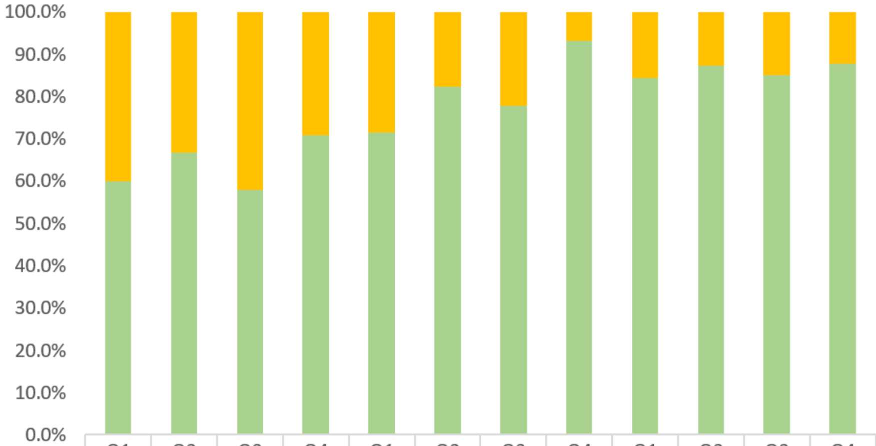

	Q1 2014	Q2 2014	Q3 2014	Q4 2014	Q1 2015	Q2 2015	Q3 2015	Q4 2015	Q1 2016	Q2 2016	Q3 2016	Q4 2016
% POST-CHEMO	40.0%	33.3%	42.1%	29.2%	28.6%	17.6%	22.2%	6.9%	15.6%	12.7%	15.0%	12.2%
% CHEMO-NAÏVE	60.0%	66.7%	57.9%	70.8%	71.4%	82.4%	77.8%	93.1%	84.4%	87.3%	85.0%	87.8%

Figure S2. Trend of chemotherapy status in ENZ users.

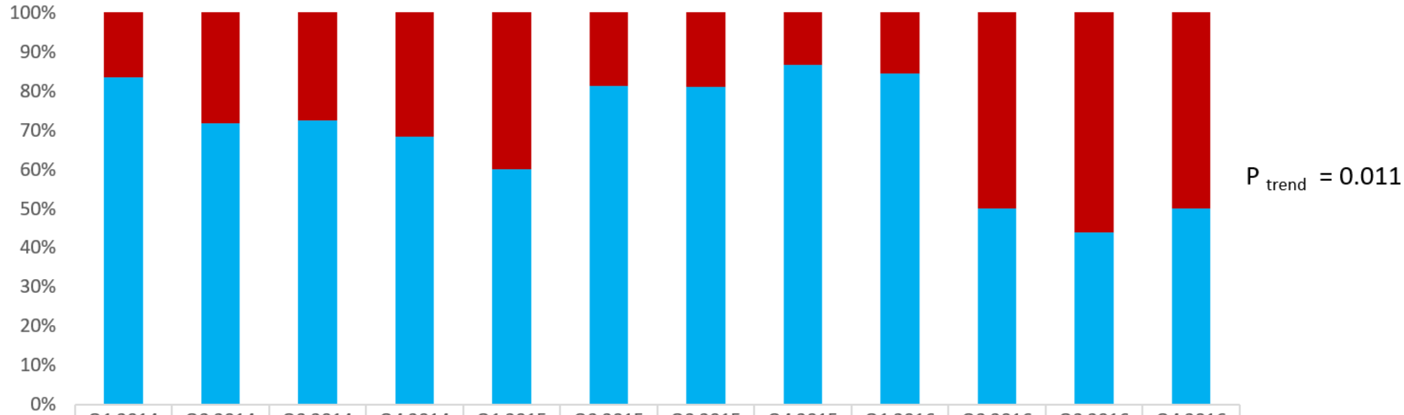

	Q1 2014	Q2 2014	Q3 2014	Q4 2014	Q1 2015	Q2 2015	Q3 2015	Q4 2015	Q1 2016	Q2 2016	Q3 2016	Q4 2016
% ENZ	17%	28%	28%	32%	40%	19%	19%	13%	16%	50%	56%	50%
% ABI	83%	72%	72%	68%	60%	81%	81%	87%	84%	50%	44%	50%

Figure S3. Trend of NHA type in post-chemotherapy patients in ENZ-era,.

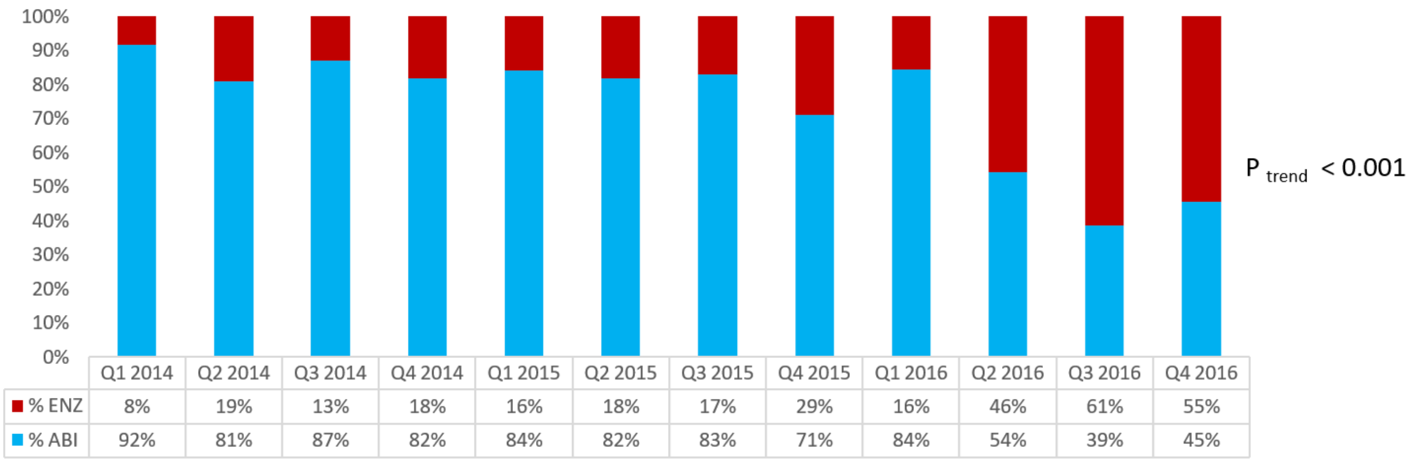

Figure S4. Trend of NHA type in chemotherapy-naïve patients in ENZ-era;.

Table S1. Description of Novel Hormonal Agent Prescription Claims.

	**Year of NHA prescription claim**					
	2011	2012	2013	2014	2015	2016
Nbr of NHA claims	133	1958	4172	6767	7796	8521
Nbr of ABI claims	133	1958	3985	5756	6339	6197
Nbr of ENZ claims	0	0	187	1011	1457	2324
Prescribed daily dose (PDD) represented by claim						
ABI				ENZ		
ABI PDD 1000mg	95.2%			ENZ PPD 160mg		94.9%
ABI PDD 750mg	1.8%			ENZ PPD 120mg		1.8%
ABI PPD 500mg	0.8%			ENZ PPD 80mg		1.6%
ABI PPD other	2.1%			ENZ PPD other		1.7%
